# RNA Detection in Live Bacterial Cells Using Fluorescent Protein Complementation Triggered by Interaction of Two RNA Aptamers with Two RNA-Binding Peptides

**DOI:** 10.3390/ph4030494

**Published:** 2011-03-10

**Authors:** Hung-Wei Yiu, Vadim V. Demidov, Paul Toran, Charles R. Cantor, Natalia E. Broude

**Affiliations:** 1 Program of Molecular and Cellular Biology and Biochemistry, Center for Advanced Biotechnology and Department of Biomedical Engineering, Boston University, 36 Cummington Street, Boston, MA 02215, USA; 2 Sequenom, Inc., 3595 John Hopkins Court, San Diego, CA 92121, USA

**Keywords:** RNA aptamer, fluorescent protein, RNA-binding viral peptide, protein complementation, RNA localization, *E. coli* cells

## Abstract

Many genetic and infectious diseases can be targeted at the RNA level as RNA is more accessible than DNA. We seek to develop new approaches for detection and tracking RNA in live cells, which is necessary for RNA-based diagnostics and therapy. We recently described a method for RNA visualization in live bacterial cells based on fluorescent protein complementation [1-3]. The RNA is tagged with an RNA aptamer that binds an RNA-binding protein with high affinity. This RNA-binding protein is expressed as two split fragments fused to the fragments of a split fluorescent protein. In the presence of RNA the fragments of the RNA-binding protein bind the aptamer and bring together the fragments of the fluorescent protein, which results in its re-assembly and fluorescence development [1-3]. Here we describe a new version of the RNA labeling method where fluorescent protein complementation is triggered by paired interactions of two different closely-positioned RNA aptamers with two different RNA-binding viral peptides. The new method, which has been developed in bacteria as a model system, uses a smaller ribonucleoprotein complementation complex, as compared with the method using split RNA-binding protein, and it can potentially be applied to a broad variety of RNA targets in both prokaryotic and eukaryotic cells. We also describe experiments exploring background fluorescence in these RNA detection systems and conditions that improve the signal-to-background ratio.

## 1. Introduction

Recent advances in RNA studies *in vivo* mostly rely on fluorescent detection methods, where RNA-specific fluorescent probes are either delivered to cells (e.g., molecular beacons) or are synthesized inside the cell (e.g., the enhanced green fluorescent protein, EGFP, fused to an RNA-binding protein, e.g., MS2 coat protein or U1Ap RNA binding protein) [4,5]. Recently, an alternative system employing lambda N peptide-GFP fusion has been described [6] and used in combination with the MS2 labeling system to simultaneously label several RNAs [7]. Notwithstanding significant progress achieved in RNA studies using these methods, they all have certain limitations, and therefore new probes and methods for labeling RNA *in vivo* are in great demand.

Protein complementation (PC) and particularly fluorescent PC is widely used for protein-protein interaction studies both *in vivo* and *in vitro* due to several advantages over the competing techniques [4,8,9]. This method allows for lower background, since split fragments of the fluorescent protein are less fluorescent than the intact protein and reassemble into fully active proteins with the help of additional forces provided by the interacting molecules. Reassembly of the fluorescent protein takes place in a narrow set of conditions and with great dynamic range that results in high signal/background ratio [8]. These features of protein complementation make them perfect for both protein and nucleic acid studies [1-3,10-14].

Recently, we described a method for RNA visualization in live cells that uses a combination of fluorescent protein complementation and high affinity interaction of an RNA-binding protein with an aptamer [1-3]. In this method, the RNA-binding protein is the eukaryotic initiation factor 4A (eIF4A) which is a two-domain RNA helicase. This large protein (about 400 amino acid residues) is dissected into two fragments corresponding to the two domains, and each fragment is fused to the fragments of the split enhanced green fluorescent protein (EGFP). Co-expression of the two fusion proteins and a transcript tagged with an eIF4A-specific aptamer resulted in the fluorescent signal in bacteria which depended on the presence of RNA target. Importantly, LacZ mRNA and 5S ribosomal RNA displayed different localization patterns consistent with their functions [1]. This suggested that the protein labeling complex is not interfering with RNA localization and function. In a modified version of the eIF4A-based method with inducible RNA synthesis, we measured RNA kinetics in single cells and visualized RNA patterns suggesting non-random RNA distribution [3].

Here we present another approach to RNA visualization in live cells based on fluorescent protein complementation. Two different RNA aptamers are added as tags to the RNA molecule of interest; they bind with high affinity two RNA-binding peptides (∼20 amino acid residues) fused with the fragments of a split EGFP (Figure 1).

There are several reasons for attempting these modifications. First, the ribonucleoprotein complex which is assembled on the target RNA is smaller as compared with that containing reassembled eIF4A protein. Smaller detection tools should provide lower probability of interference with function. Second, eIF4A protein is a component of the translation machinery in eukaryotic cells and it also has close homologs among bacterial proteins. Therefore, it cannot be excluded that its over-expression may interfere with essential biological functions in live cells. Short viral peptides on the other hand have no homologs in bacterial or in eukaryotic cells. Therefore their expression in a cell will less likely interfere with the cellular functions. Finally, an alternative design of the RNA recognizing complex adds more flexibility to the PC approach and expands the set of RNA-binding molecules applicable for RNA labeling methods.

## 2. Experimental

### 2.1. Gel-Shift Assay of Peptide-RNA Aptamer Complexes Formed In Vitro

Custom-synthesized peptides, HTLV-1 Rex, HIV-1 Rev and bacteriophage λN, were purchased from GeneScript, (Piscataway, NJ) (Table 1). RNA aptamers as well as all DNA primers and templates were from IDT (Coralville, IA) (Table 1s of the Appendix). To assure correct folding of RNA aptamers, RNAs were first denatured by heating at 95 °C in buffer containing 50 mM Tris-HCl (pH 8.0), 50 mM KCl for 3 min and then slowly cooled to room temperature. Re-folded RNAs were mixed with increasing concentrations of the peptides in the same buffer and incubated at 30 °C for 15 min. Peptide-RNA aptamer complexes were analyzed by gel-shift assay using 15% polyacrylamige gel electrophoresis in Tris-Borate-EDTA buffer and then stained with ethidium bromide.

### 2.2. Cloning and Expression of RNA Aptamers and Protein Fusions

The EGFP gene was split between amino acid residues 158 and 159 into two fragments, termed α-EGFP (N-terminal fragment) and β-EGFP (C-terminal fragment), respectively. A multi-step PCR was performed to create a DNA fragment that encodes two fusion proteins, HTLV-1 Rex peptide-α-EGFP and bacteriophage λN peptide-β-EGFP, plus T7 promoter in between (the resulting plasmid and the detailed PCR scheme are shown in Figure 1s and Figure 2s of the Appendix). HTLV-1 Rex peptide was fused to the C-terminus of the α-EGFP via a flexible 10-mer oligopeptide linker (Gly-Ser-Ser-Gly-Ser-Ser-Gly-Ser-Gly-Ser); bacteriophage λN peptide was fused to the N-terminus of the β-EGFP via the same 10-mer linker. The DNA construct was inserted into the pACYCDuet-1 vector (Novagen) between the restriction sites *NcoI* and *Avr*II, which placed the insert after the first T7 promoter region and before the T7 terminator region (Figure 1s). All constructs were verified by sequencing. Thus, two fusion proteins were expressed from the two-cystronic pACYCDuet-1 plasmid to ensure similar amounts of both proteins. Expression of both fusion proteins was verified by protein gel electrophoresis.

To clone aptamers, a DNA sequence was designed which encodes two RNA aptamers, one of which binds the λN peptide and the other the HTLV-1 Rex peptide. The aptamer sequences were separated by a dT_10_ linker, and the restriction sites for *Xba*I and *Avr*II were added at the ends. Custom synthesized DNA template was PCR-amplified, inserted between the *Xba*I and *Avr*II restriction sites in the pETDuet-1 vector (Novagen) under the control of the T7/*LacO* promoter, and cloned in *E. coli* strain BL21 (DE3). As a result, RNA was transcribed from this plasmid as a short untranslated transcript without a ribosome binding site and containing two adjacent aptamers separated by 10Ts. As a negative control, DNA sequence encoding two identical RNA aptamer sequences binding HTLV-1 Rex peptides was synthesized and similarly cloned into pETDuet-1 vector. DNA templates and primers are listed in Table 1s and Table 2s of the Appendix.

### 2.3. Cell Growth Conditions and Induction

Two plasmids encoding protein and RNA components of the complementation complexes were co-expressed in *E. coli* BL21 (DE) 3 *(B F^−^ dcm ompT hsdS (r_B_^−^ m_B_^−^) gal λ* (DE3)) (Stratagene). As a negative control, the cells were transformed with a plasmid expressing two identical aptamers. Single colonies of transformed cells were grown first at 37 °C in LB medium supplemented with antibiotics for 3 h. Then, cultures were diluted 300-fold into fresh LB medium containing the inducer isopropyl-β-D-thiogalactopyranoside (IPTG) and grown overnight at room temperature. Optical density of the cultures (OD_600_) was between 0.4 and 0.6 at the time of examination.

### 2.4. Flow Cytometry

Fluorescence measurements were obtained with a Becton-Dickinson FACSCalibur flow cytometer with a 488-nm argon excitation laser and a 515–545 nm emission filter (FL1). 500 μL of the cells induced overnight (OD_600_ = 0.4 to 0.6) were washed once with PBS buffer prior to assaying. In total 100,000 cells have been analyzed.

### 2.5. Fluorescence Microscopy, Imaging and Data Analysis

Induced bacterial cells in culture were immobilized between a cover slip and a thin slab of 0.8% agarose in 1X PBS. Time-lapse fluorescence microscopy was performed at room temperature with a Nikon Eclipse 80i inverted microscope equipped with an epifluorescence system X-Cite 120. Images were taken with exposure times of 150–300 ms using a digital black and white camera (12 bit; 20 mHz) with 100x magnification objective controlled by IPLab v.3.7 software (Scanalytics, Inc). An ND4 filter was used to reduce cell photo-damage. Pseudo-green color was added according to the fluorescence level in the florescent images. Image processing was performed using ImageJ 1.36 B software (Wayne Rasband, NIH).

## 3. Results and Discussion

### 3.1. Design of a Protein Complementation Complex for RNA Detection Based on Interaction of the Two RNA Aptamers with Two RNA-Binding Viral Peptides

To detect RNA in live bacterial cells we tagged it with two different RNA aptamer sequences which bind two different viral peptides with high affinity. Each peptide was expressed in the cell as a fusion with one of the two fragments of a split enhanced green fluorescent protein (EGFP) (Figure 1). Interaction of each peptide with its cognate aptamer should bring together the two fragments of split EGFP. High local concentrations of the split fragments of EGFP will result in their re-association and development of the fluorescent signal. This approach is a further elaboration of a PC method used by Rackam and Brown to study RNA/protein interactions [14]. In this study, an MS2 binding motif was artificially introduced into RNA, while the second RNA/protein contact was probed by PC. To do so the authors expressed MS2 protein and RNA-binding proteins (FMRP or IMP1) as fusions with the split fluorescent proteins. In other words in this study one aptamer was introduced into RNA, while the other site was an endogenous RNA site which interaction with the RNA-binding protein was in question [14].

To develop an efficient PC method based on two aptamers/two peptides interactions, several issues should be considered. The affinity of each interacting peptide/aptamer pair should be high enough (in the nanomolar range) and ideally it should be comparable for both pairs. There should be no cross-reactivity between the two peptide/aptamer pairs. Also, there should be no interaction between the RNA-binding peptides in absence of RNA, which could otherwise bring the fragments of a detector protein together and thus increase the non-specific background. The RNA-binding peptides should be of comparable length to avoid distorting the assembly of the protein complementation complex. Finally, the placement of the two RNA aptamers on the target RNA should allow accessibility of the corresponding RNA-binding peptides to the cognate RNA aptamers. This implies that a flexible linker of sufficient length should be placed between the RNA aptamer tags.

It is known that the high-affinity and high-specificity interactions of many RNA-binding proteins with the corresponding RNAs are determined by the peptide sequences which contain arginine-rich motifs (ARMs) [15]. Recent studies aimed to understand the mechanism of ARM peptides interaction with the corresponding RNAs concluded that specific binding is determined by a particular pattern of arginines in the peptide and flexibility of the peptide backbone [16]. However, many ARM peptides display a promiscuous behavior by binding several different RNA targets, although with lesser affinity [17]. Keeping all this in mind, we chose three RNA-binding peptides from viral ARM peptides [18-20], and first tested their cross-reactivity with the corresponding RNAs before employing them in our protein complementation system.

### 3.2. *In Vitro* Testing of Cross-Reactivity of RNA Aptamer-Peptide Pairs

In these *in vitro* experiments the increasing concentrations of the ARM peptides were combined with RNA aptamers at fixed concentrations. The complexes were then qualitatively analyzed by a non-radioactive gel-shift assay (see Figure 2 for the exemplary gel picture). The results showed that the bacteriophage λN peptide and HTLV-1 Rex peptide bind their corresponding RNA aptamers in the nanomolar concentration range and did not display cross-reactivity with the non-cognate aptamers. At the same time, HIV-1 Rev peptide did show some cross-reactivity with the two non-matched aptamers (Figure 2 and Table 2). Based on these results, we concluded that the bacteriophage λN and HTLV-1 Rex peptides along with their corresponding RNA aptamers can be used in the PC-based RNA detection method.

### 3.3. Optimization of Detection of RNA Transcripts in Live Bacterial Cells Using Binary Peptide/Aptamer Interactions

The C-terminus of the EGFP fragment (1–158 aa) was fused to the N-terminus of the HTLV-1 Rex peptide (16 aa-long) via a flexible linker consisting of serine and glycine residues [21,22]. We used the same GS-rich linkers that we used earlier in our eIF4A-based complementation system [1-3]. Similarly, the N-terminus of the second EGFP fragment (159–238 aa) was fused to the C-terminus of λN peptide (22 aa) via the same polypeptide linker. The vector pETDuet-1 (Novagen) was used for the expression of an untranslated T7-transcript containing two aptamer sequences linked by the T_10_ sequence. Preliminary experiments with T_5_ and T_10_ linkers did not show substantial difference. Therefore, we used the constructs with T_10_ linkers through out this study.

*E. coli* cells expressing the entire complementation complex and appropriate controls were grown overnight at room temperature in the presence of the inducer, isopropyl-ß-D-thiogalactopyranoside (IPTG) for co-expression of proteins and RNA. Fluorescence of these cells was compared with fluorescence of the cells expressing two protein fusions in RNA absence and cells expressing two protein fusions plus an incorrect combination of the two aptamers (two HTLV-1 Rex peptide-binding aptamers linked with the T_10_-linker).

**Figure 3:**
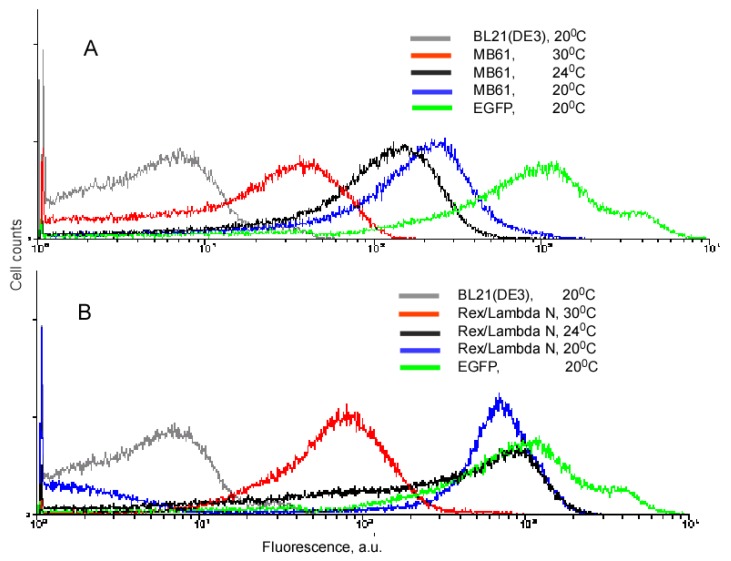
Background fluorescence in *E. coli* cells expressing protein fusions can be regulated by the temperature of cell culturing. (**A**). *E. coli* MB61 cells express two fusion proteins α-F1 and β-F2, fragments of EGFP and eIF4A proteins. Two fusion proteins were cloned in the vector pACYCDuet 1 and induced with 1 mM IPTG at different temperatures. Fluorescence of the *E. coli* cells BL21(DE3) and the cells expressing intact EGFP from the same vector is shown for comparison; (**B**). Rex/Lambda N denote *E. coli* cells which express two fusion proteins containing fragments of split EGFP fused to HTLV-1 Rex and lambda N peptides. The protein fusions were cloned into the plasmid pACYCDuet 1 and induced with 1 mM IPTG at different temperatures. Fluorescence of the *E. coli* cells BL21(DE3) and the cells expressing intact EGFP is shown for comparison.

Our experiments with the complementing fusion proteins containing short viral peptides, as well as the fragments of the split eIF4A protein, revealed that background fluorescence caused by spurious self-assembly of the protein fragments can be modulated by cell growth conditions and IPTG concentration (Figure 3). These two parameters, the temperature and IPTG concentration, have an effect on the split protein concentrations by two different mechanisms. Low IPTG concentrations decrease the overall concentration of split proteins, and thus effectively lower the incidence of their spurious re-assembly. The temperature of overnight culture incubation also has a large effect on cell fluorescence by affecting the concentration of properly folded split proteins. At lower temperature, the amount of properly folded and functional proteins synthesized is higher than at higher temperature. Therefore, at 20 °C a high incidence of spurious re-assembly results in a higher background signal than at 30 °C (∼100–200 a. u. versus 20–40 a. u.), in the system based on the eIF4A protein (Figure 3A). It should be emphasized that this background is two orders of magnitude lower than in the cells expressing native EGFP (Figure 3). The cells expressing fusions of the split EGFP with the viral peptides usually displayed higher background than the cells expressing split eIF4A fusion proteins (compare Figures 3A and B). This can probably be explained by the positive charges of these arginine-rich peptides that non-specifically bind to the negatively charged molecules or surfaces. To overcome this non-specific background in the cells expressing fusion proteins with the viral peptides we changed the second parameter affecting signal-to-background ratio: we varied concentrations of the inducer, IPTG.

**Figure 4:**
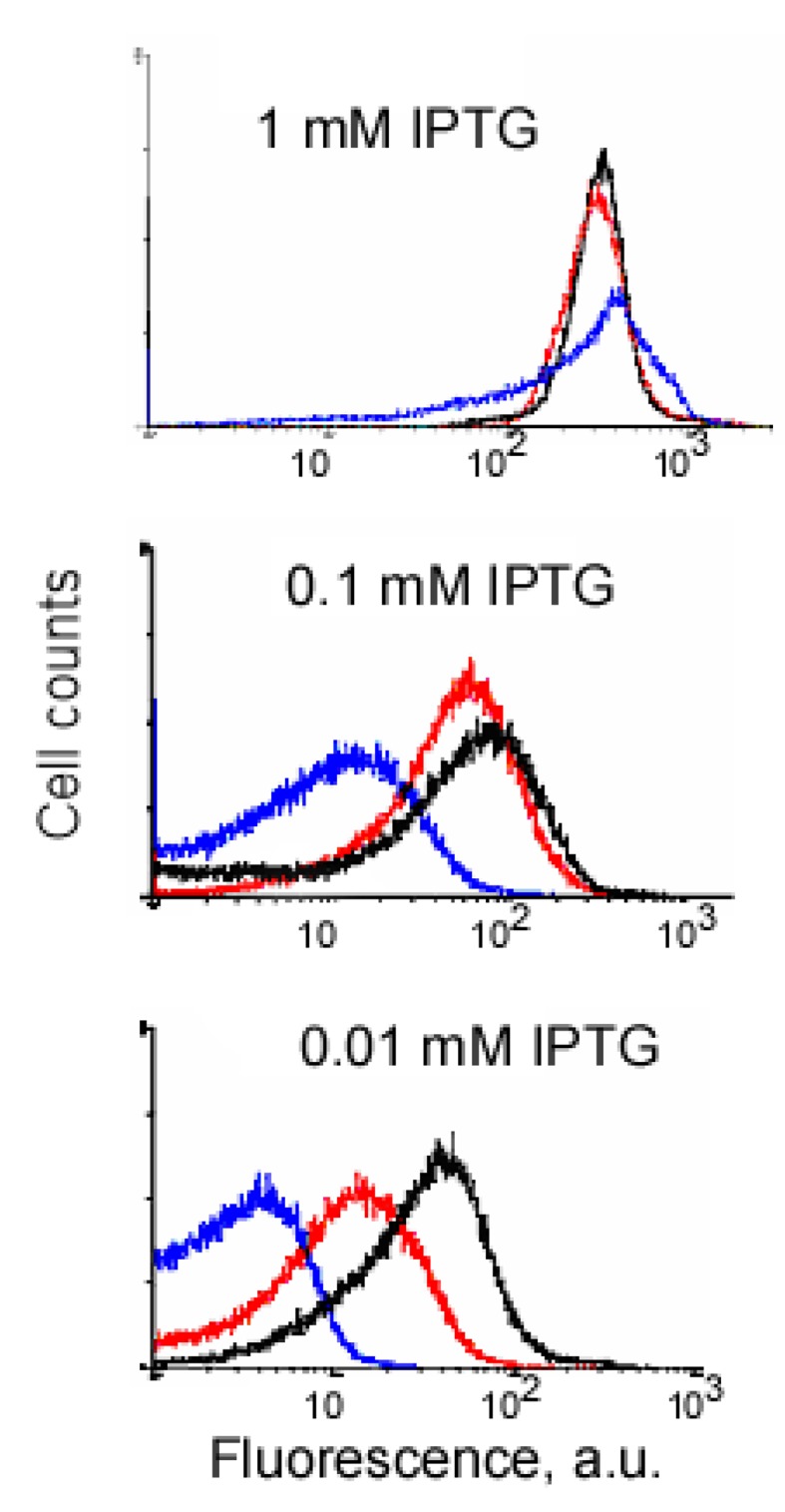
Low concentration of IPTG allows discrimination of signal from the background using binary aptamer/peptide interactions. Blue, fluorescence distributions of the cells expressing two fusion proteins in RNA absence; red, fluorescence distribution of the cells expressing two fusion proteins and RNA aptamers with the wrong sequences (two HTLV-1 Rex aptamers); black, fluorescence distribution of the *E. coli* cells expressing two fusion proteins and RNA with two cognate aptamers.

**Figure 5:**
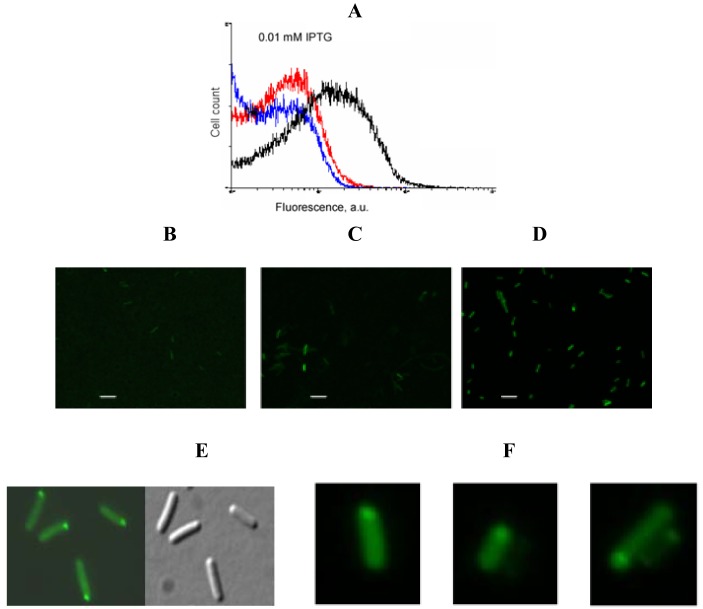
Fluorescent images of *E. coli* cells expressing RNA transcript tagged with the two aptamers. (**A**). FACS analysis of the *E. coli* cells: Blue, the cells express two fusion proteins in RNA absence; red, the cells express two fusion proteins and RNA aptamers with the wrong sequences (two HTLV-1 Rex aptamers); black, *E. coli* cells express two fusion proteins and RNA with two cognate aptamers; (**B**). Fluorescent microscopy: The control cells express fusion proteins in RNA absence; (**C**). the control cells express RNA with one non-cognate aptamer and fusion proteins; (**D**). the cells express fusion proteins and a transcript with two cognate aptamers. Scale bar, 5 μm; all images were taken under similar conditions; (**E**). Fluorescence distributions in single *E. coli* cells expressing reporter RNA and labeled using eIF4A system [1]; (**F**). Fluorescence distributions in single *E. coli* cells expressing reporter RNA and labeled using binary viral peptide/aptamer system.

An example of signal-to-background optimization experiments with the protein complementation system using viral peptides is shown in Figure 4. At 25 °C *E. coli* cells expressing two fusion proteins induced with 1 mM IPTG displayed high fluorescence in the absence of RNA target, and there was also no difference in fluorescence distribution in the cells expressing correct or incorrect aptamer sequences (Figure 4). Decreasing the concentration of IPTG 10-fold resulted in separation of the fluorescence distributions for the cells expressing only fusion proteins and those expressing fusion proteins and RNA aptamers (Figure 4). By decreasing the concentration of IPTG to 0.01 mM it was possible to resolve the cognate aptamer-dependent fluorescence from that of the targets with incorrect aptamer sequences (Figure 4). Under optimized conditions, the average fluorescence of the cells expressing the entire complementation complex exceeded background fluorescence (no RNA component) 10–15 fold, and cells with correct RNA-tagging aptamer sequences displayed 4–5 times higher fluorescence than cells with the non-matched RNA tags (Figure 4 and Figure 5).

Bacterial cells expressing a short untranslated RNA tagged with the two aptamers at optimized conditions were analyzed using fluorescent microscopy. In most cells bright fluorescent spots were seen at the cell poles (Figure 5F) similar to the results obtained in the experiments when an untranslated transcript was labeled by PC triggered by eIF4A-aptamer interactions [1] (Figure 5E).

## 4. Conclusions

In this study we aimed at development of an alternative to eIF4A protein-based fluorescent complementation system which would widen the choice of molecules for RNA labeling in live cells. The results show that the RNA detection system based on binary interactions of two RNA aptamers with short viral peptides presents an attractive alternative to the system based on the split eIF4A protein. It uses smaller protein complementation complex consisting of short viral peptides which are less likely to interfere with bacterial or eukaryotic cell metabolism. We have shown that under optimized conditions when the two viral peptides recognize two correct RNA aptamers the mean fluorescence of the cells exceeds negative controls 3–4 fold, as it was the case with the eIF4A-based system [1].

The experiments exploring background fluorescence showed that in both protein complementation systems (using split eIF4A or two short viral peptides) the source of background fluorescence is the same and it can be modulated by changing conditions of cell growth, specifically by concentrations of the inducer, IPTG, and by changing the temperature of cell culturing. The slightly higher background in the PC system using viral peptides can be likely explained by the aggregation of the arginine-rich peptides. However, it does not change the major trend characteristic to both PC systems (compare the results in Figures 3A and B). We should also emphasize that the presented results are important not only for the RNA PC-based studies, but for all methods using fluorescent PC.

## Figures and Tables

**Figure 1:**
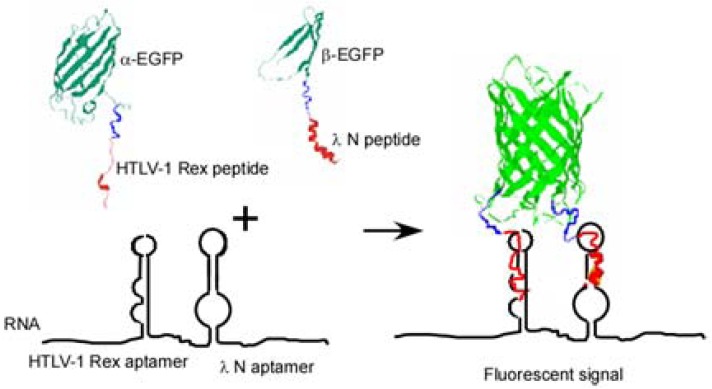
Fluorescent protein complementation based on binary peptide-RNA aptamer interactions. Two fragments of EGFP, α- and β-, are fused with the two viral peptides, HIV-1 Rex peptide and bacteriophage λN peptide. In the presence of an RNA transcript bearing two corresponding aptamers, the two peptides interact with their cognate aptamers and bring together two fragments of split EGFP. Re-assembly of EGFP results in fluorescent signal.

**Figure 2:**
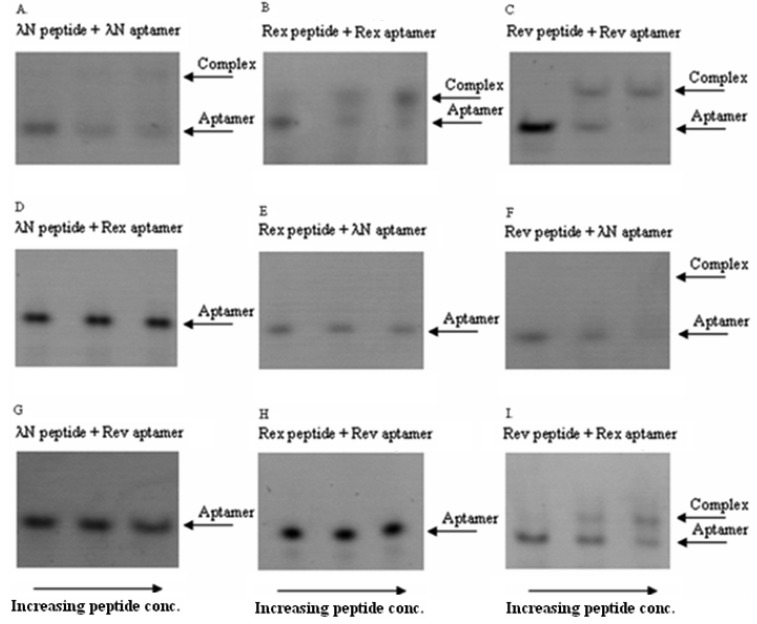
Cross-reactivity of three pairs of peptide/RNA aptamers. In these titration experiments, the variable amounts of viral peptides were in excess over fixed amounts of RNA aptamers (∼20 nmole).

**Table 1:** Peptide and RNA aptamer sequences used in this study.

**Peptide/aptamer pair**	**Bacteriophage λN/aptamer pair**	**HTLV-1 Rex/aptamer pair**	**HIV-1 Rev/aptamer pair**
RNA aptamer sequence in MFOLD format	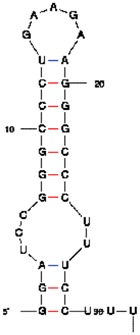	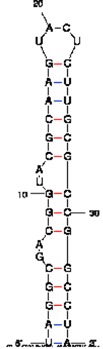	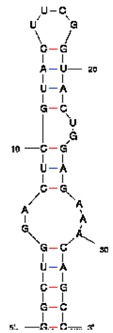
Peptide sequence	MDAQTRRRERRAEKQAQWKAAN	MPKTRRRPRRSQRKRP	TRQARRNRRRRWRERQR
Reference	[20]	[18]	[19]

**Table 2:** Cross-reactivity between three RNA aptamer/peptide pairs. (++, strong interaction; +, cross-reactivity; −, lack of interaction).

**RNA aptamer**	**HTLV-1 Rex**	**λN**	**HIV-1 Rev**
**Peptide**			
HTLV-1 Rex peptide *	++	−	−
Bacteriophage λN peptide	−	++	−
HIV-1 Rev peptide	+	+	++

* 50% binding of HTLV-1 Rex peptide to HTLV-1 Rex aptamer was observed at a concentration of peptide ∼300 nM, when this peptide was in excess over RNA.

**Figure 1s:**
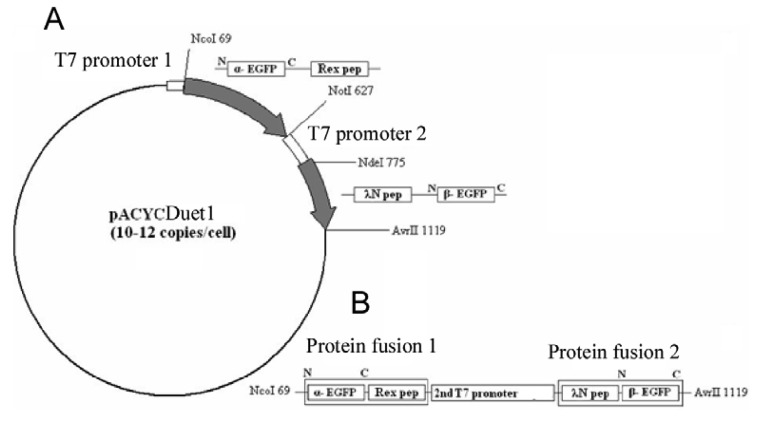
The plasmid expressing two fusion proteins. (**A**). Plasmid structure; (**B**). DNA fragment that was created by multi-step PCR (see Figure 2s) and inserted into vector pACYCDuet-1 between NcoI /AvrII sites.

**Figure 2s:**
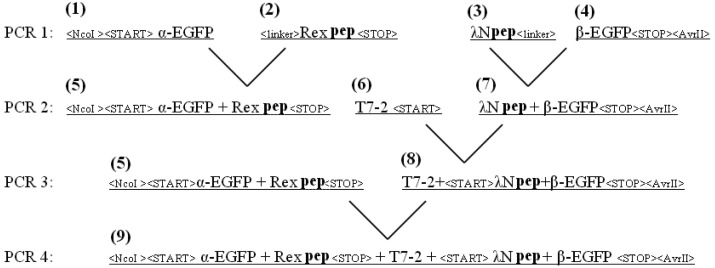
Multi-step PCR used to obtain DNA fragment encoding the two fusion proteins and all regulatory regions Each pair of PCR products to be joined has about 10 bp of sequence overlap. Pairs were mixed and used as a template for the next PCR reaction. The plasmid containing EGFP gene (from Clonetech) was used as a template for PCR products (1) and (4). Templates for PCR products (2), (3) and (6) were custom synthesized. After PCR 1, PCR products (1) and (2) were mixed and used as a template for amplifying PCR product (5). PCR products (3) and (4) were mixed and used as a template for amplifying PCR product (7). After PCR 2 amplification, PCR products (6) and (7) were mixed as a template for amplifying PCR product (8). The last PCR 4 was carried out by mixing PCR products (5) and (8) as a template. Final PCR product (9) was inserted into pACYCDuet-1 vector. Sequences of DNA templates and primers are listed in Table 1s and Table 2s.

## Appendix

**Table 1s:** DNA templates used in this study for PCR amplifications.

**Template Encode**	**Sequence (5′ to 3′)**
Linker + Rex peptide	GGATCCTCTGGATCTTCGGGTTCTGGTAGCATGCCCAAGACCCGT CGGAGGCCCCGCCGATCCCAAAGAAAAAGACCA
λN peptide + linker	ATGGATGCACAAACACGCCGCCGCGAACGTCGCGCAGAGAAACAGGCTCAATGGAAAGCAGCAAATGGATCCTCTGGATCTTCGGGTTCTGGT AGC
Rex and λN aptamers + linker	CACCAGGCCACGAGTCTAGAGGATCCGGGCCCTGAAGAAGGGCCCTTTCCTTTTTTTTTTTTTTAGGCGACGGTACGCAAGTACTCTTGCGCCGGCCTACCTAGGGACGAGCAGCAGGC
2 Rex aptamers + linker	CACCAGGCCACGAGTCTAGATAGGCGACGGTACGCAAGTACTCTTGCGCCGGCCTATTTTTTTTTTTAGGCGACGGTACGCAAGTACTCTTGCGCCGGCCTACCTAGGGACGAGCAGCAGGC

**Table 2s:** Primers used for PCR amplification (see Figure 2s).

**Primer**	**Sequence (5′ to 3′)**	**Note**
P1UP	ATA ACC CAT GGT GAG CAA GGG C	Upstream primer for PCR products (1)(5)
P1DN	CAG AGG ATC CCT GCT TGT CGG CC	Downstream primer for PCR product (1)
P2UP	CGA CAA GCA GGG ATC CTC TGG ATC T	Upstream primer for PCR product (2)
P2DN	TGC GGC CGC TCA TGG TCT TTT TCT T	Downstream primer for PCR products (2)(5)
P3UP	AAA AAG ACC ATG AGC GGC CGC ATA ATG	Upstream primer for PCR products (6)(8)
P3DN	CGT GTT TGT GCA TCC ATA TGT ATA TCT CCT T	Downstream primer for PCR product (6)
P4UP	GAT ATA CAT ATG GAT GCA CAA ACA CGC	Upstream primer for PCR products (3)(7)
P4DN	TGC CGT TCT TGC TAC CAG AAC C	Downstream primer PCR product (3)
P5UP	TTC TGG TAG CAA GAA CGG CAT CAA G	Upstream primer for PCR product (4)
P5DN	TCA AGC CTA GGT TAC TTG TAC AGC TC	Downstream primer for PCR products (4)(7)(8)(9)
AptUP	CAC CAG GCC ACG AG T CTA GA	Upstream primer for PCR of the aptamer gene
AptDN	GCC TGC TGC TCG TCC CTA GG	Downstream primer for PCR of the aptamer gene
